# Estimating sliding drop width via side-view features using recurrent neural networks

**DOI:** 10.1038/s41598-024-62194-w

**Published:** 2024-05-27

**Authors:** Sajjad Shumaly, Fahimeh Darvish, Xiaomei Li, Oleksandra Kukharenko, Werner Steffen, Yanhui Guo, Hans-Jürgen Butt, Rüdiger Berger

**Affiliations:** 1 Max Planck Institute for Polymer Research (MPI-P), Ackermannweg 10, 55128 Mainz, Germany; 2 Department of Computer Science, University of Illinois Springfield, Springfield, IL USA

**Keywords:** Sliding drops, Drop width estimation, Multivariate sequence analysis, Recurrent neural network (RNN), Long short-term memory (LSTM), Gated recurrent unit (GRU), Bidirectional LSTM (BiLSTM), Convolutional neural network (CNN), Convolutional LSTM (ConvLSTM), Wetting, Characterization and analytical techniques

## Abstract

High speed side-view videos of sliding drops enable researchers to investigate drop dynamics and surface properties. However, understanding the physics of sliding requires knowledge of the drop width. A front-view perspective of the drop is necessary. In particular, the drop’s width is a crucial parameter owing to its association with the friction force. Incorporating extra cameras or mirrors to monitor changes in the width of drops from a front-view perspective is cumbersome and limits the viewing area. This limitation impedes a comprehensive analysis of sliding drops, especially when they interact with surface defects. Our study explores the use of various regression and multivariate sequence analysis (MSA) models to estimate the drop width at a solid surface solely from side-view videos. This approach eliminates the need to incorporate additional equipment into the experimental setup. In addition, it ensures an unlimited viewing area of sliding drops. The Long Short Term Memory (LSTM) model with a 20 sliding window size has the best performance with the lowest root mean square error (RMSE) of 67 µm. Within the spectrum of drop widths in our dataset, ranging from 1.6 to 4.4 mm, this RMSE indicates that we can predict the width of sliding drops with an error of 2.4%. Furthermore, the applied LSTM model provides a drop width across the whole sliding length of 5 cm, previously unattainable.

## Introduction

Researchers have employed side-view video recordings of sliding drops to analyze the physico-chemical behavior of the liquid to solid interface surface^1,2^. Investigation of the wealth of phenomena of sliding drops requires a measurement of the drop shape from the front-view^3–5^. Often extra cameras or mirrors are added for front-view analysis. This approach, while common, brings practical challenges. In continuing, we will present two examples highlighting the significance of drop width and the need for front-view observation.

**Figure 1:**
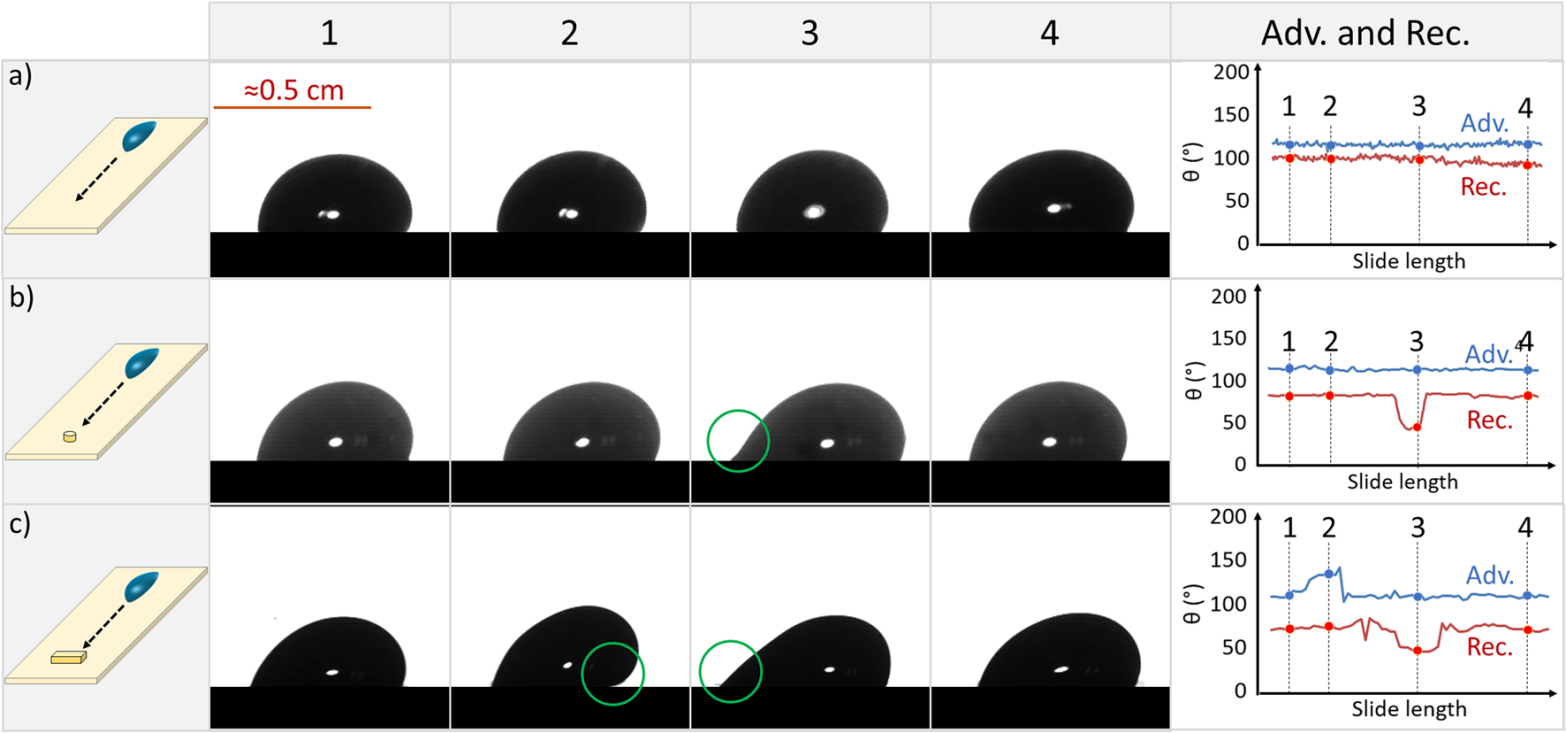
Side-view images of 32 µL water drops at different positions along their path. (**a**) The Thiols_Au sample has no large defects or heterogeneity. The tilt angle was 30°. (**b**) The PFOTS_Si sample has a single cylindrical defect (D-Cy-800), with a height of 31 µm, which affects the dynamic receding contact angle (${\theta }_{r}$) of the drop (green circle). The dynamic advancing contact angle (${\theta }_{a}$) is not changed significantly. The tilt angle was 35°. (**c**) The PFOTS_Si sample has a block defect (D-Bl-3000), with a height of 174 µm, that affects both dynamic advancing and dynamic receding contact angles considerably due to its larger size (two green circles). The tilt angle was 50°. By increasing the defect size, the tilt angle is increased to allow the drop to pass the defect. In the specific examples presented above, we only plotted ${\theta }_{a}$ and ${\theta }_{r}$, but additional parameters of the drop such as drop length, velocity, drop height, and middle line angle changes as well.

On homogeneous surfaces, hydrodynamic dissipation increases with velocity. Recently, Li et al. studied drops sliding down an inclined surface^3^ and reported an empirical equation that describes the velocity-dependent friction force:

1 $${F}_{f}= {F}_{0}+\beta wU\eta$$

where, ${F}_{0}$ is the friction force extrapolated to velocity $U$= 0, $\beta$ is a dimensionless friction coefficient, $w$ is the width of the drop while sliding, and $\eta$ is the viscosity of the liquid. The geometry of drops and their kinetic contact angles sliding down an inclined plane change with velocity. These parameters can be readily measured in a side-view (Fig. 1). However, within a standard sliding drop experiment, determining the drop width while sliding remains a challenging task.

In addition to homogeneous surfaces, topographical and chemical variations spanning from nano- to micro-meters on surfaces lead to the pinning of the three-phase contact line^6^. Pinning of the contact line increases contact angle hysteresis (CAH), and drop friction, thus decreases drop velocity. The interactions between solid surfaces and liquids are described by the liquid–air surface tension ($\gamma )$,  the width of the contact area (*w*), and the apparent rear and front contact angles of the drop (${\theta }_{a}$, and ${\theta }_{r}$)^4,5^:

2 $${F}_{LA}=k\gamma w(cos{\theta }_{r}-cos{\theta }_{a})$$

where ${F}_{LA}$ is the lateral adhesion force, ${\theta }_{a}$ is the advancing angle, ${\theta }_{r}$ is the receding angle, and $k$ is the geometry factor^7^. The lateral adhesion force has been related to external forces that cause a drop to slide, such as gravitational^8^, drag by a micropipette^9^, and centrifugal^10^ forces. Equation (2) is often called the Furmidge equation and was first reported in the 40ies^11–14^. The Furmidge equation indicates that the drop width is directly related to lateral adhesion force. The Furmidge equation was also taken to calculate ${F}_{LA}$ for sliding drops. Interestingly, the calculated ${F}_{LA}$ is consistent with forces calculated by the equation of motion^3^ assuming *k* = 1. Thus, measuring ${\theta }_{a}$, ${\theta }_{r}$ and $w$ will allow us to calculate ${F}_{LA}$.

However, determining the drop width of sliding drops is challenging. Drop width data can be collected by recording bottom-view or front-view videos of sliding drops. Bottom-view imaging is restricted to transparent substrates. Front-view imaging of drops over a sliding length of ≈1.5 cm is feasible by installing a second, time-synchronized high-speed camera^15^. Also, a second high-speed camera can be omitted by installing two mirrors at the back and front of the drop sliding length^16^. One mirror reflects light from the light source and via the other front-view videos are recorded. The primary challenge in the experiment lies in optimizing lighting conditions for the additional mirrors/second camera. Positioning mirrors in the optical path is tricky, and can lead to a reduced contrast in the final front-view images due to light reflection. Introducing another camera necessitates an extra front-view illuminator, doubling the equipment on a platform that must rotate 90°. More importantly, in both cases, the direction of drop motion towards the camera or mirrors limits the focus area for front-view images to approximately 1.5 cm, significantly narrowing the observable area.

The question we faced was whether we could estimate drop width based solely on side-view measurements. The sliding behavior of a drop involves changes in the dynamic advancing (${\theta }_{a}$) and receding (${\theta }_{r}$) angles, drop length, velocity, drop height, and middle line angle. These factors can interact in complex ways, creating intricate patterns. For drops sliding on homogeneous surfaces, ${\theta }_{a}$ and ${\theta }_{r}$ change monotonically throughout the entire sliding path, as shown exemplarily for a perfluorodecanethiol monolayer on gold coated glass (Thiols_Au) sample (Fig. 1a). In cases where an obstacle is encountered along the drop's route, alterations to the dynamic contact angles occur. For a cylindrical obstacle with a diameter of 800 µm (D-Cy-800), we measured that ${\theta }_{r}$ changes while only minimally influencing the ${\theta }_{a}$ on a 1H,1H,2H,2H-perfluoroctyltrichlorosilane coated silicon wafer sample (PFOTS_Si, Fig. 1b). For a bigger block defect, 800 µm thick and 3000 µm length (D-Bl-3000), on a PFOTS_Si surface, both the ${\theta }_{a}$ and ${\theta }_{r}$ change considerably (Fig. 1c).

Machine learning models were successfully deployed in surface science. In a study on water drop impact on supercooled surfaces, two models were developed to predict the spreading dynamics of the drop upon impact and classify the resulting icing patterns^17^. These models aimed to forecast the degree of surface supercooling corresponding to the observed icing patterns. DeepAngle is a machine learning-based approach, to accurately determine contact angles in tomography images of porous materials^18^. Traditional methods for measuring 3D angles face challenges in voxelized spaces. Therefore, deep learning models were applied to estimate interfacial angles directly from images, instead of computationally intensive grid-based approaches. The latter enhances accuracy to 16% while reducing computational costs 20-fold. Tanaka et al., developed a neural network model, which allows prediction of contact angles for a wide range of metals and oxide surfaces^19^. A CNN-based method was applied for contact angle measurement of the moving drops to overcome traditional algorithm limitations due to optical distortions by changes in the focal length^20^. The proposed method exhibits robustness against higher Gaussian Blurring values. Recently, we presented a CNN-based super-resolution technique that achieved a more precise analysis of sliding drops^21^. The reported approach led to a 21% increase in accuracy for contact angles below 90° and a 33% improvement for contact angles above 90°.

Here, we explore different machine learning models for estimating drop width based on drop parameters recorded dynamically from the side-view. Our contributions are outlined as follows:

1. We introduce a machine learning-based approach. This approach does not require additional mirrors or cameras traditionally used for a frontal view of the sliding drop. The machine learning-based approach significantly simplifies the experimental setup.
2. For the first time, our machine learning-based approach enables the continuous monitoring of a sliding drop along its entire path, which typically is 5 cm. The viewing area is not limited by the type of optics. This capability overcomes limitations that restrict observations to the final centimeter of a sliding drop.
3. We conducted a comparative analysis between regression models and multivariate sequence analysis (MSA) models. This comparison delves into the independence or interdependence of drop parameters relative to its preceding and subsequent values. With this comparison, researchers are able to select the most appropriate model to meet their specific needs.

In the “Materials and Methods” section, we delve into the cutting-edge methods currently used for sequence analysis. We describe in detail the experimental procedures employed to collect the dataset, and prepare the relevant samples. Then we describe the dataset's structure and explain our training process. In the “Results and Discussion” section, we evaluate the precision of various regression and MSA (Multivariate Sequence Analysis) algorithms in predicting drop width. We further authenticate the performance of our optimal model using a sample with different defect height. Additionally, we perform sensitivity analysis to equip researchers with the necessary insights to effectively apply our developments in their investigations. The developed LSTM model and its updates is accessible in the GitHub repository^22^.

## Materials and methods

### Sequence analysis methods

Many real-world prediction problems have been successfully addressed using deep learning methods, including time-series forecasting^23,24^. Recurrent neural network (RNN) is a deep learning time series analysis method that has been gaining significant interest recently^25–27^. RNNs enhance the accuracy of predictions by using their recurrent architecture, which allows them to capture and utilize sequential information and temporal dependencies in data. The effectiveness of RNNs stems from their capacity to incorporate past events and their utilization of shared parameters or weights. Each RNN cell takes an input and combines it with the previous hidden state to produce an output and a new hidden state.

However, deep neural networks with a fully connected architecture are prone to the problem of vanishing gradient^28^. A vanishing gradient occurs when the network is unable to update its weights due to its activation functions and network architectures, which cause gradient values to be too small during backpropagation^29^. RNN is a biased model and gives high importance to recent occurrences, reducing its effectiveness^30^. Thus in applications, RNN is refined into RNN-based models, such as Long Short-Term Memory (LSTM) and Gated Recurrent Units (GRU) models.

A solution for the vanishing gradient problem is a method called LSTM^31,32^. The fundamental concept underpinning the LSTM architecture involves a memory cell with the ability to sustain its state throughout time. Coupled with nonlinear gating units, these components oversee the inflow and outflow of information from the cell^33^. Each LSTM cell has three gates: a forget gate, an update gate, and an output gate. The LSTM network architecture can learn from past data using its gates in order to remember the past data and thus creates a prospective model based on the past and current data. In this way, LSTM models can capture sequence pattern information more efficiently^31^. In LSTM, the forget gate's role is to determine which data from the previous cell state should be eliminated for the current time step. While, the update gate is responsible for selecting what new information is eligible for storage in the cell state, and the output gate manages the information available for output based on the cell state.

The Gated Recurrent Unit (GRU) emerges as an innovative approach aiming to streamline the complexity inherent in LSTM^34^. GRU, like LSTM, utilizes gating techniques to regulate the network’s information flow selectively. GRU only uses the reset gate and the update gate, as opposed to LSTM's three gates. The update gate regulates the combination of incoming input with the old state, whereas the reset gate regulates the extent to which the past information is ignored. GRU is more computationally efficient owing to this streamlined architecture, which has fewer parameters than LSTM^35^. Supporting information (SI-RNNs’ architectures) includes the RNN, LSTM, and GRU architectures as well as their formulas.

The Bidirectional LSTM (BiLSTM) model is an advancement of LSTM and involves two LSTM cells, namely the forward and backward LSTMs^36^. In BiLSTM, the input sequence is processed twice, first from left to right and then from right to left. The LSTM takes into account all previous events, whereas the BiLSTM considers both past and future events. BiLSTMs are thus superior to LSTMs in some cases^37^.

The field of computer vision has been revolutionized by convolutional neural networks (CNNs)^38^. CNN was successfully applied in various fields and researchers used it for time series analysis^39^. In general, CNNs can handle spatial auto-correlated data, detecting patterns in short-term, and time series data with local dependencies. However, they are not typically trained to handle long temporal relationships^40^. Therefore, a time-series model which exploits the benefits of both CNNs and LSTMs, such as Convolutional LSTM (ConvLSTM), will be able to capture also long-term dependencies ^41–43^.

A number of models have been proposed to tackle the challenge of interpreting deep models and identifying important features ^44–46^. Gradient-weighted Class Activation Mapping (Grad-CAM) is an interpretation model that was originally designed for image processing^47^. Grad-CAM is versatile and is extended for text and sound analysis^48–50^. Similar to Grad-CAM, which calculates the gradients of a target output with respect to convolutional layer activations to identify important regions in an image, we used gradient-based feature importance to assess the influence of input features on the output of the LSTM model.

### Data gathering

**Figure 2:**
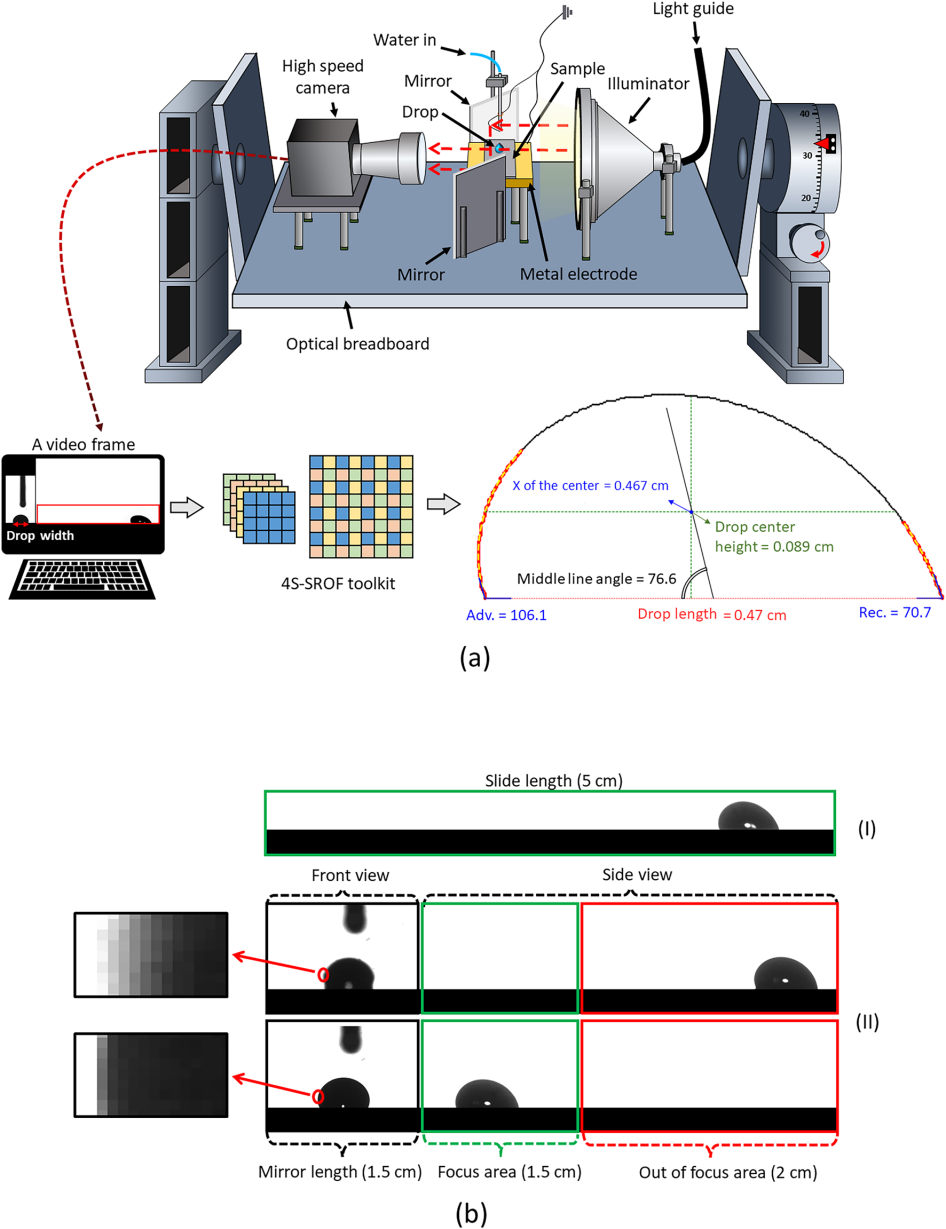
(**a**) A sketch of the tilted plane experimental setup. Traces of sliding drops were recorded by a high-speed camera equipped with a telecentric objective. Videos were stored on a computer. Drop contact angles, drop length, drop center height, median line angles, and velocity were extracted by a 4S-SROF toolkit. (**b**) Snapshot of sliding drop in side- and front-view. (**I**) One frame from a video which was recorded without mirrors. Here the slide length for the drop is ≈5 cm. (**II**) The mirrors that are required to image a drop in front-view occupy ≈1.5 cm of the camera field of view. Furthermore, the initial ≈2 cm of the video will be out of focus in the front view, as demonstrated exemplarily by magnifying the interface between the liquid and the air. The use of the mirror reduces the effective area for recording the sliding length of the drop from ≈5 to ≈1.5 cm.

Drops of distilled water (< 1 µS cm^–1^; Gibco, Thermo Fisher Scientific) with a volume of 32 µl were placed on top of a tilted plane using a peristaltic pump (MINIPULS 3, Gilson) connected to a grounded, blunt syringe needle (1.5 mm outer diameter) (Fig. 2a). The liquids were dropped from a height of approximately 5 mm, which enabled them to detach from the syringe before reaching the surface. The optical breadboard with mounted components can be rotated from 0 to 90°. By rotating the entire setup the alignment of the optical setup is kept^16^. In particular, the angle between the video camera and the sample stays constant close to 0°. Videos of sliding drops were recorded with a high-speed camera (FASTCAM Mini UX100, Photron) equipped with a TitanTL telecentric lens (× 0.268, one inch, C-mount, Edmund Optics). The front-view of the sliding drops was captured at the same time by reflecting the backlight from the telecentric backlight illuminator (Edmund Optics) using two parallel mirrors (25 × 36 mm^2^ protected silver mirror; PFR10-P01, Thorlabs) on both sides of the sample. The temperature and humidity during the experiment were approximately 20 ± 1 °C and 15–30%, respectively. A standard backlight illuminator was used limiting frame rates to 500 fps. At higher frame rates the contrast of images reduces and the drop shape cannot be analyzed.

We employed the 4-segment super-resolution optimized-fitting (4S-SROF)^21,51^ toolkit to extract drop velocity, drop center height, drop length, ${\theta }_{a}$, ${\theta }_{r}$ and the drop’s middle line angle from the recorded videos (Fig. 2a). The velocity is calculated based on the position of the center of the drop (X of the center in the figure). All experiments were repeated several times at different tilt angles and analyzed by the 4S-SROF toolkit, resulting in our dataset (SI-Data distribution). It was essential to gather data during changes in the sliding drop pattern. For instance, when changing the surface chemistry, the drop velocity was altered. Surface defects were introduced, to increase the model's generality. Also, in produced samples the height of the defects changes, and we varied the viscosity of the liquid by using mixtures of water and glycerol of varying concentrations. We used DI water with a viscosity of 0.92 mPa s, 20% glycerol-water mixture (1.7 mPa s), 30% glycerol-water mixture (2.5 mPa s), and 40% glycerol-water mixture (3.8 mPa s).

### Sample preparation

Samples with block and cylindrical defects were produced on silicon wafers. First, wafers were cleaned by ultrasonication in acetone twice, followed by 2-propanol to remove organic impurities. Next, cleaned Si-wafers were exposed to an O_2_ plasma (140 W, 5 min, 0.3 mbar, Femto–Diener GmbH, Germany). A filtered nitrogen gun that blows away microscopic fibers and dust was used to dry and remove the small particles from the Si-wafers. Then, 1 ml SU-8 (either GM1060, or GM1070, Gersteltec Sàrl, Switzerland) was dropped on Si-wafers. In the GM1060 case, the rotation speed was ramped up from 500 rpm (7 s) to 1000 rpm (40 s) for the validation block defect. In the GM1070 case, the rotation speed was ramped up from 500 rpm (7 s) to 700 rpm (40 s), from 500 rpm (7 s) to 1000 rpm (40 s), and from 500 rpm (7 s) to 1500 rpm (40 s) for different block defects. Also, in the GM1060 case, the rotation speed was ramped up from 500 rpm (7 s) to 850 rpm (40 s), and from 500 rpm (7 s) to 600 rpm (40 s) for different cylindrical defects. To evaporate solvents from the thin layers of resist, a soft baking process was carried out on a hotplate (65 °C for 30 min, 95 °C for 2–4 min, and 65 °C for 30 min). After gradually cooling down to room temperature, the samples were mounted to a mask aligner (MJB3, Süss Microtec, Germany). UV light through a photomask exposed for 8 s with 290 J/cm^2^ energy. After that, post-exposure baking for 1–2 min at 95 °C was performed. Unpolymerized SU-8 was removed by rinsing with 1-methoxy-2-propanol acetate (CAS# 108-65-6). Finally, samples are washed for 1 min in 2-propanol. Before fluorination, samples were activated by the oxygen plasma for 5 min 140 W, 0.3 mbar. 0.5 mL of 1H,1H,2H,2H-perfluoroctyl trichlorosilane (PFOTS, 97%, CAS:78,560-45-9, PFOTS) was selected due to its low boiling point of 180 °C, which was beneficial for easy evaporation, and its high reactivity for deposition. Fluorination was performed in a CVD chamber under 40–50 mbar pressure at room temperature for 30 min. To remove uncrosslinked PFOTS, the samples were kept at 0 mbar for 30 min and then washed and rinsed with ethanol and Milli Q water. Also, defect-free PFOTS_Si samples were produced using the given procedure.

To prepare samples with gold-coated glass substrates (gold substrate), 5 nm Chromium and 35 nm gold were sputter coated onto glass substrates subsequently (BalTec MED 020). The gold substrates were used immediately and directly without further cleaning. Thiols-gold samples were prepared by submerging gold substrates in a 1 mM ethanolic 1H,1H,2H,2H-perfluorodecanethiol (≥ 96.0%; Sigma-Aldrich) solution for 24 h. Before using, we rinsed the samples with absolute ethanol to remove unbounded thiols and dried them with air blowing. To get Teflon-gold samples, ~ 60 nm Teflon film was coated on the gold substrate by dip coating with a pulling speed of 10 mm/min from a solution of 1wt% Teflon AF 1600 (ε = 1.9; Sigma-Aldrich) in FC-75 (97%, Fisher Scientific). Finally, the Teflon samples were annealed in a vacuum oven at 160 °C for 24 h before use.

### Data structure and training process

Without the use of mirrors in our tilted plane setup, the field of view covers a sliding length of 5 cm (Fig. 2b_I). The installation of mirrors cut the field of view of the camera; The front-view image with a field of view of ≈1.5 cm and the side-view with a length of ≈3.5 cm (Fig. 2b_II). Please note, when the drops are situated in the initial 2 cm of the sample, they are out of focus of the telecentric objective due to the large distance from the reflecting mirror. Only in the last ≈1.5 cm of the slide path drops are in focus.

The dataset consists of side-view images with their corresponding front-view images. The initial 2 cm of sliding length was excluded from the dataset, since precise drop width measurement was not possible. Thus, the initial 2 cm sliding length was not used for training models. Defects were fabricated on the last cm of samples, where we capture the drop shape in the side- and front-view.

The dataset was filtered to include only videos with more than 20 and less than 250 frames, aiming to ensure data consistency and relevance. Min–max scaling was utilized to standardize every aspect of the dataset within a range spanning from negative one to positive one. This process ensures that all features are on a similar scale, preventing any feature from dominating the analysis due to its magnitude. The data was segmented into smaller windows of 5, 10, 15, and 20 consecutive frames, with overlap, using a sliding window approach. This segmentation facilitates the analysis of sequential data and allows the model to capture temporal patterns effectively.

Our dataset consisted of 235 videos, including 13,301 frames. Each video contains different number of frames. The dataset was divided into two distinct subsets. A testing subset was created comprising 10% of the dataset (hold-out testing dataset), while the remaining 90% was allocated for training, in all cases.

We employed a grid search algorithm^52^ to fine-tune the critical hyperparameters of each regressor, aiming to identify the optimal configuration as follow:

- **Multilayer perceptron:**
  - Number of layers: Varies from 1 to 4 with intervals of 1.
  - Number of nodes: Varies from 25 to 100 with intervals of 25.
  - Alpha values: Options include 0.01, and 0.1.
  - Learning rates: Options include 0.001, 0.01, and 0.1.
- **Gradient boosting:**
  - Number of estimators: Varies from 50 to 300 with intervals of 50.
  - Minimum samples split: Options include 2, 5.
  - Maximum depth: Options include none, 5, or 10.
  - Minimum samples leaf: Options include 1, 2.
  - Learning rates: Options include 0.01, 0.1, and 0.2.
- **Random forest:**
  - Number of estimators: Varies from 50 to 300 with intervals of 50.
  - Maximum features: Options include 'sqrt', 'log2'.
  - Maximum depth: Options include none, 5, and 10.
  - Minimum samples split: Options include 2, 5.
  - Minimum samples leaf: Options include 1, 2.

In the case of MSA models, to prevent overfitting during training, a validation set was created by taking 20% of the training data. The validation set allowed monitoring of the model's performance on unseen data during the training process. This separation ensures that the model's performance can be evaluated on unseen data during the training process. The number of epochs for MSA models was set to 2500, as this was found to be the point at which the loss diagrams began to plateau. The observation of a plateau ensured that the model was able to converge to an optimal solution without overfitting the training data. Given the time-intensive nature of the training process, we bypassed the grid search algorithm for tuning MSA hyperparameters. Instead, we explored a single-layer LSTM configuration, experimenting with unit sizes of 32, 48, 64, and 128 to achieve a satisfactory structure. We employed a consistent structure across all models, involving a single layer with 48 units, whether it was LSTM, GRU, BiLSTM, or ConvLSTM. Additionally, a dropout rate of 0.5 and L2 kernel and recurrent regularization of 0.01 were consistently applied across all cases to prevent overfitting. The activation function employed was “tanh”, and the optimizer used was “adam” for all MSA model configurations. This approach allowed for a balanced comparison of model performance under standardized conditions.

We utilized the mean square error (MSE, Eq. 3) as the loss function for MSA models. The MSE calculates the average of the squared differences between the predicted values and the actual target values. MSE is commonly employed for sequence analysis. In addition, we use root mean square error (RMSE, Eq. 4) and mean absolute error (MAE, Eq. 5) formulas to evaluate the accuracies:

3 $$MSE= \frac{{\sum }_{i=1}^{n}{({x}_{i}-\widehat{{x}_{i}})}^{2}}{n}$$

4 $$RMSE= \sqrt{\frac{{\sum }_{i=1}^{n}{({x}_{i}-\widehat{{x}_{i}})}^{2}}{n}}$$

5 $$MAE= \frac{{\sum }_{i=1}^{n}\left|{x}_{i}-\widehat{{x}_{i}}\right|}{n}$$

where ${x}_{i}$ is the true value, $\widehat{{x}_{i}}$ is the estimated value, and $n$ is the total number of data points.

The choice of RMSE over MSE for representing the error results was motivated by RMSE’s capability to retain the same unit as the original data. Utilizing RMSE enhances the ease of understanding and interpretation of the results. In this case, the error is calculated based on the unit of drop width, which is “μm”. The utilization of two measures is justified by the fact that MAE assesses the overall error and provides insights into the general estimation quality of the developed model. On the other hand, RMSE penalizes errors by squaring them, thereby giving importance to large errors. The inclusion of both RMSE and MAE allows for a thorough evaluation of the model's performance, encompassing both the overall estimation accuracy and the consideration of large errors.

## Results and discussion

### Drop width estimation

**Figure 3:**
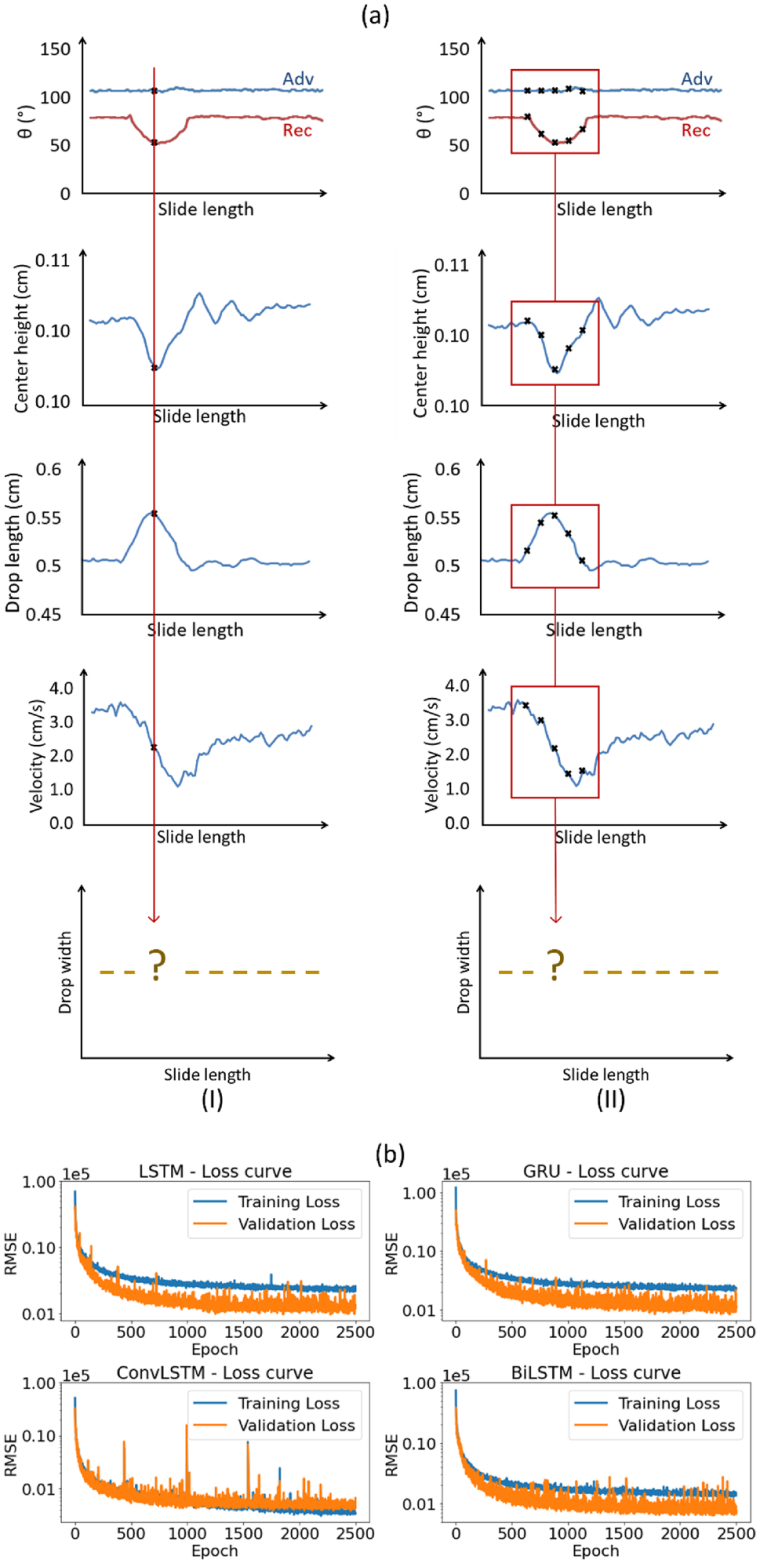
(**a**) A representation of two approaches to estimating the drop width. A regression approach that treats each observation independently (i). (**b**) An MSA approach that considers temporal dependencies by utilizing a sliding window (ii). The plotted ${\theta }_{a}$ and ${\theta }_{r}$, drop center height, drop length, and velocity are based on a real measurement of a sample with a cylindrical defect. The calculation accounts for the middle line angle, which has not been explicitly depicted here for simplicity. (**b**) Comparison of training loss curves for LSTM, GRU, ConvLSTM, and BiLSTM architectures. The curves depict the evolution of the training loss over epochs, illustrating the convergence behavior and efficiency of each model in learning the task of estimating drop width. Note that the y-axis employs a logarithmic scale, necessitating careful examination of the data.

In regression techniques, we assume that it is possible to estimate the drop width from each side-view image of a video (Fig. 3a_I). Hence, the drop width is an outcome of all features of the drop accessible from an individual side-view image. Regression techniques disregard the temporal dependencies that exist between time steps in the data. Therefore this approach is applicable for data sets where there is only a weak or negligible temporal dependency between the past and future data. To create a model that offers simplicity and interpretability, we used a linear regression model^53^. In addition to being useful for analyzing direct relationships between variables, linear regression can be utilized to assess the complexity of other models. To identify complex nonlinear correlations in the data, a multilayer perceptron model^54^ was used. The multilayer perceptron model excels in managing intricate patterns and relationships. The choice of the random forest^55^ and gradient boosting^56^ algorithms stems from the aim to improve generalization and mitigate overfitting; their robustness and versatility are utilized to achieve the enhancement.

As an alternative, it is possible to estimate the width of the drop at a given slide length by considering the shape of the drop at the previous and following slide length, i.e. previous and following images of a video (Fig. 3a_II). Thus with MSA, temporal dependencies are taken into account. By considering the temporal dependencies between time steps, a superior outcome can potentially be achieved when addressing the problem using MSA instead of regression methods. This strategy is more intricate and resource-intensive. Moreover, these methods are not as easily interpretable as regression models. We will investigate for different scenarios whether the accuracy achieved through the MSA is significantly better than that achieved through a simple regression method. We utilized LSTM and GRU architectures because they are renowned for their ability to capture both short and long dependencies. The ConvLSTM model was chosen for its noise resistance, while the BiLSTM model was selected for its bidirectional sequential information review capability.

The comparison of training loss curves across LSTM, GRU, ConvLSTM, and BiLSTM models showcases the differences in their performance over epochs (Fig. 3b). These curves demonstrate how the training loss decreases with each epoch, highlighting the models' ability to converge and efficiently learn to estimate drop width. A minimal discrepancy between training and validation losses typically indicates that the model has captured the dataset's essential patterns without memorizing the data (overfitting) or failing to learn sufficiently (underfitting). This equilibrium suggests the model's effective generalization to unseen data, reflecting a successful training process. The validation loss is slightly lower than the training loss likely due to the use of dropout during the training process.

We compared regression algorithms and MSA methods for drop sliding based on our test set. The accuracy of MSA methods was significantly better than that of regression in estimating drop width (Table 1). The best result for the regression algorithms in terms of both RMSE and MAE criteria was obtained using random forest, with values of 109.0 µm and 90.0 µm, respectively. LSTM as an MSA algorithm achieved the best result based on both criteria with values of 67.6 µm and 58.5 µm, respectively. The 67.6 µm error would be translated to about 2.4% error percentage when considering the full range of drop width values in the test dataset. The evaluation of MSA methods took place by examining the accuracies across different sliding window sizes, 5, 10, 15, and 20. Using a sliding window size of 5, for instance, we will estimate one drop width using five frames. The results indicate that increasing the sliding window size leads to accuracy improvement. These findings provide evidence that temporal dependencies play a vital role in predicting drop width.

**Table 1:** Exploring the accuracy of drop width estimation through regression and MSA.

Type of model	Model	Sliding window size (# frames)	RMSE (µm)	MAE (µm)
Regression	Multilayer perceptron	–	144.8	122.4
	Gradient boosting	–	125.0	94.4
	Random forest	–	**109.0**	**90.0**
	Linear regression	–	194.9	179.3
MSA	GRU	5	93.2	83.9
	GRU	10	83.3	75.4
	GRU	15	83.3	74.9
	GRU	20	81.7	71.9
	LSTM	5	96.5	84.8
	LSTM	10	81.4	73.8
	LSTM	15	67.7	60.5
	LSTM	20	**67.6**	**58.5**
	ConvLSTM	20	77.8	64.3
	BiLSTM	20	71.1	62.8

Significant values are in [bold].

**Figure 4:**
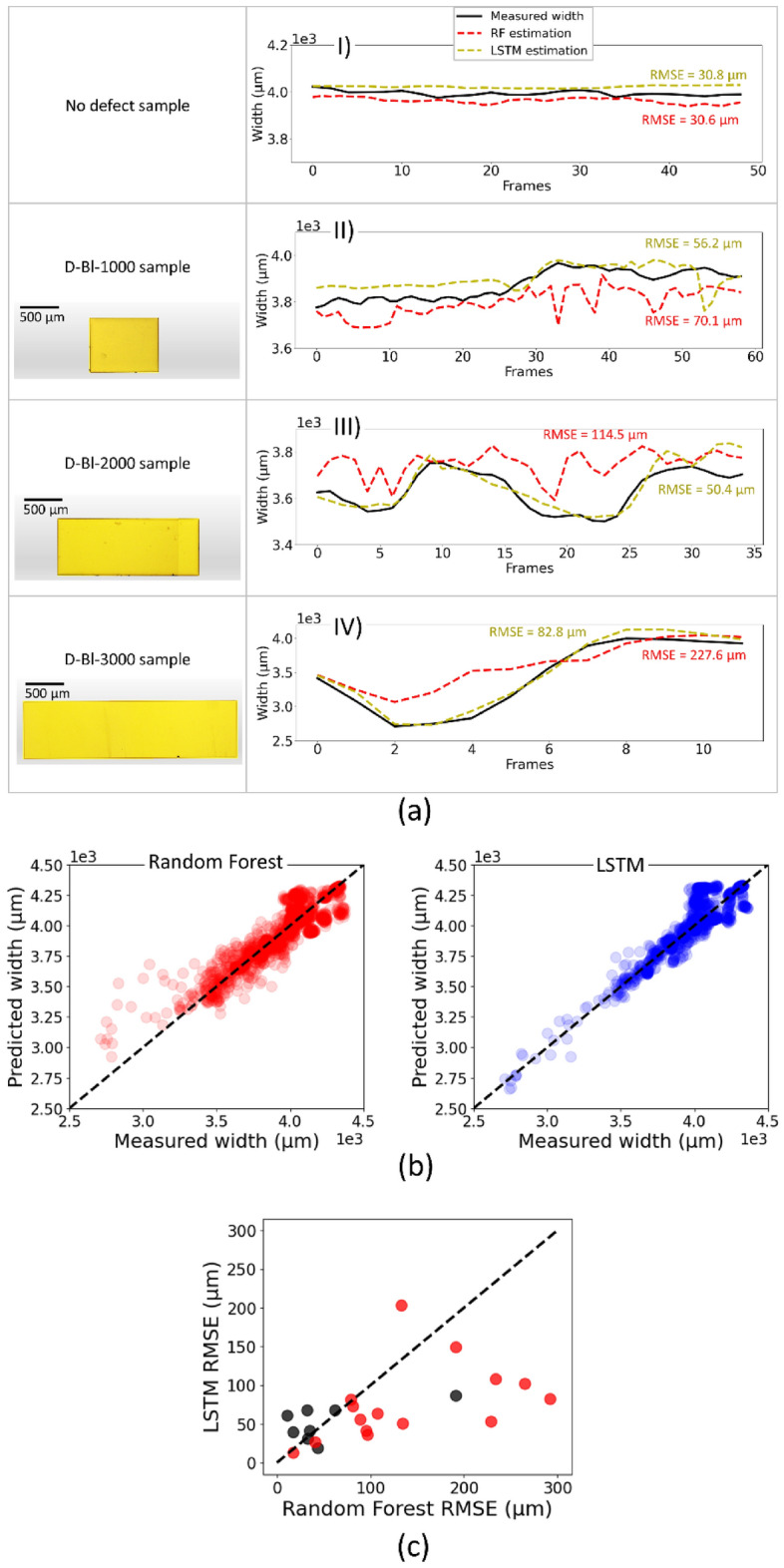
(**a**) Comparison of estimated and measured drop width. The representative examples were analyzed using both RF and LSTM (with 20 sliding window size) models on samples with and without defects. One sample without defect, and three samples with block defects, D-Bl-1000 (thickness = 800 μm, length = 1000 μm, and height = 106 μm), D-Bl-2000 (thickness = 800 μm, length = 2000 μm, and height = 74 μm), D-Bl-3000 (thickness = 800 μm, length = 3000 μm, and height = 174 μm), respectively. The drop width on the last ≈1.5 cm of the sliding movement is analyzed. In all cases defect microscopic image has been illustrated in the left column. (**b**) LSTM and RF predictions vs. real measurements on the entire test dataset based on frames. (**c**) Accuracy of LSTM vs. RF predictions on entire test dataset based on videos. The red dots represent the RMSE related to videos of sliding drops on surfaces containing defects. Also, black dots represent the same for surfaces without defects.

The main differences between random forest (RF) and LSTM with a sliding window size of 20 frames have been explored by visualizing their respective estimations on a small subset of the test dataset (Fig. 4a). For the sliding of a drop on a sample without defect and the D-Bl-1000 sample, the estimation accuracy of RF was found to be close to that of LSTM (Fig. 4a I, II**)**. However, with an increase in the length of the block defect, the accuracy of RF gradually diminished (Fig. 4a III, IV**)**. When estimating the drop width on samples with block defects D-Bl-2000 also D-Bl-3000, the RF achieved RMSE of 114.5 μm and 227.6 μm, while LSTM attained RMSE of 50.4 μm and 82.8 μm, respectively.

The diagrams that were discussed represented a subset of the test data. To assess the accuracy of the algorithms across the entire test dataset, we considered the measured/estimated drop width. We plot the measured width against the predicted value for every frame in the test dataset (Fig. 4b). The plot indicates that the LSTM model yields superior results compared to the RF model for frames where the drop width becomes < 3.5 µm. These widths are present in cases where drop interacts with the surface defects. The dense clustering of scatter plot points around the line of identity in the LSTM graph indicates enhanced prediction accuracy for drop widths.

To better understand why RF is not good at predicting drop width in the presence of defects, we compared results obtained with LSTM's RMSE and that of RF (Fig. 4c). We separated videos of sliding drops on surfaces containing defects (red dots) and videos of sliding drops on surfaces without defects (black dots). It turned out that on samples without defects, the accuracy of both methods is similar. On samples with defects, LSTM is better.

The primary cause for a larger error in the RF model is its failure to precisely predict the drop's width while it is interacting with defects. This failure arises from regression models’ failure to account for occurrence dependencies, resulting in the omission of crucial information. However, the RF results outperformed other regression algorithms due to the dataset's imbalance. This implies that the occurrence of defects is an infrequent event. The RF, as a bagging method, exhibits a reasonable level of robustness to imbalanced data by itself^57,58^. Nevertheless, for research studies of the dynamic behavior of drops on samples without defects^3^ RF provides accurate width values. Even a simple linear regression with the below formula works on the defect-free part of our test dataset with 95.8 RMSE for 32 µl drops.

6 $$Drop width=-351.7 \times {\theta }_{a} + 411.6\times {\theta }_{r}+ 118.4\times {\text{D}}.{\text{L}}.- 188.0\times {\text{D}}.{\text{C}}.{\text{H}}. - 841.4\times {\text{Vel}}. + 850.6\times {\text{M}}.{\text{L}}.{\text{A}}.+ 3351.9$$

while ${\theta }_{a}$ is advancing angle, ${\theta }_{r}$ is receding angle, D.L. is drop length, D.C.H. is drop center height, Vel. is velocity, and M.L.A. is middle line angle.

### Validation of a new sample

To validate our model, we produced a sample of PFOTS_Si containing a block defect, thickness = 800 μm, length = 3000 μm, and height = 23 μm. There were no videos related to this specific defect in neither the training nor testing dataset. The large size of this defect for the LSTM model poses the greatest difficulty in terms of prediction among all defects we considered. The defect of the new sample has a height of 23 μm, a dimension not included in the model's training data (as per SI-Data distribution). We intend to test this specific sample with defect to evaluate the model's ability to generalize. Following the same procedures as before, mirrors were employed to directly measure the drop width, allowing for a precise assessment of the prediction accuracy. As a result of mirror installation, the side-view visible slide length decreased to ≈3.5 cm.

**Figure 5:**
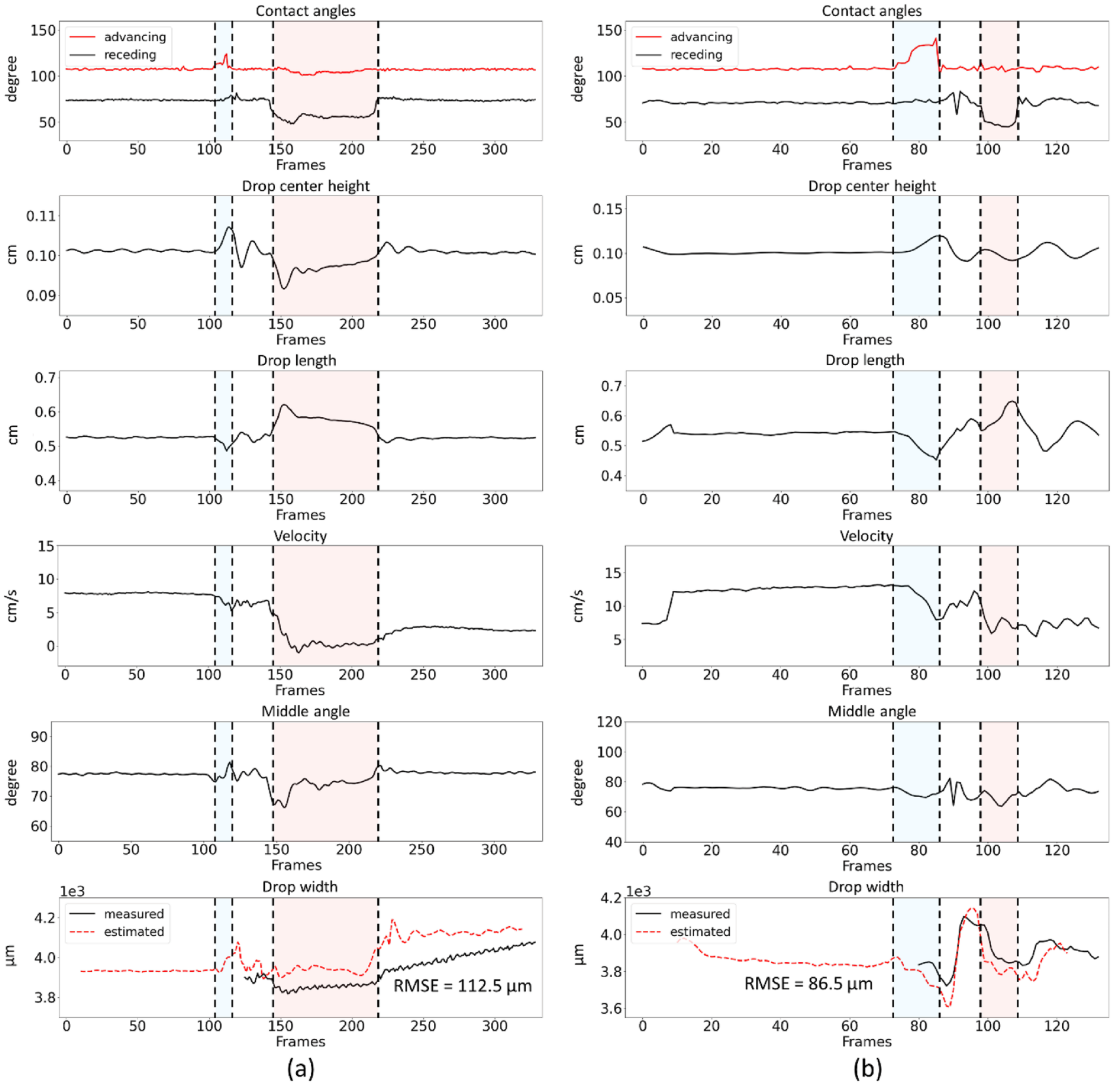
All side-views extracted measures and the estimated vs. measured drop width diagrams for drop sliding at (**a**) 42° and (**b**) 45° tilt angles. To showcase that the developed model is not restricted to the final ≈1.5 cm of sliding motion, the estimation of drop width was displayed for ≈3.5 cm of the slide (red dotted diagrams). The blue region represents when the advancing part was stuck to the defect. The red region represents when the receding part was stuck to the defect. The recording speed was 500 fps. The drop volume was 32 µl. In drop width estimating using LSTM with 20 sliding window size, we would lose the first 10 frames and the last 9 frames due to the sliding window size.

We employed the obtained measurements to estimate the drop width through the developed LSTM model with sliding window size of 20 frames. It was only possible to directly measure the drop width, as done before, for the last ≈1.5 cm of sliding motion. This experiment shows that the developed model is not restricted to the final ≈1.5 cm of sliding motion, and the estimation for block defects is displayed for ≈3.5 cm of the slide. The drop width was measured and estimated for sliding drops at tilt angles of 42° (Fig. 5a) and 45° (Fig. 5b).

The tilt angle of 42° was the lowest angle at which the drop could slide and pass the defect. Thus, drop velocity was slow and the receding contact line pinned to the defect for a while (Fig. 5a, the red region of diagrams). By increasing the tilt angle, the drop velocity increased and the drop did not pin. The advancing contact angle was more affected than the lower tilt angle (Fig. 5b, the blue region of diagrams). Also, drop center height, drop length, and middle angle degree measures were used to estimate the drop width. The drop width estimation accuracy for videos of sliding drop at 42° was 112.5 µm, and at 45° was 86.5 µm.

### Sensitivity analysis

**Figure 6:**
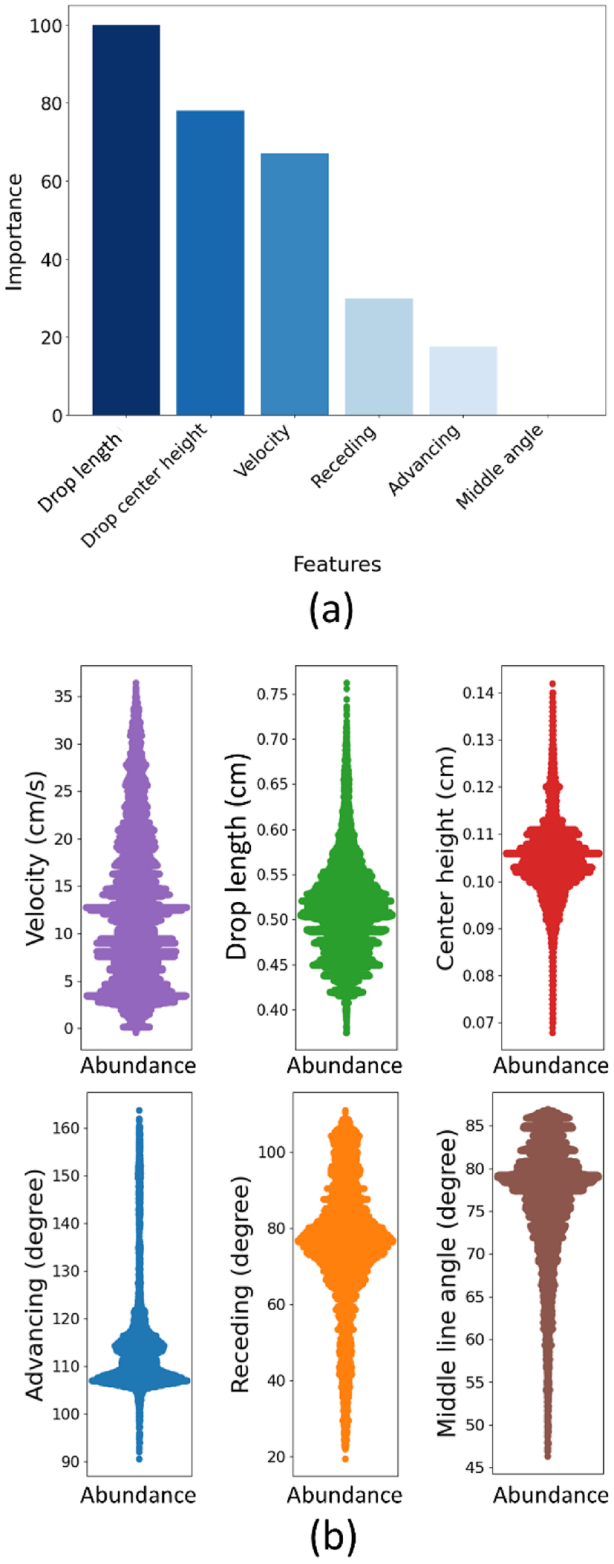
(**a**) Analysis of LSTM feature importance based on the Grad-CAM Method. The results show that drop length, drop center height, and velocity are the most important features in estimating drop width. The importance is normalized between 0 and 100. (**b**) The distribution of observations in the training data based on features. Each point represents an existing frame in the dataset, related to a particular value on the y-axis.

To ascertain which variable(s) exert the greatest impact on the estimation of drop width, we carried out a feature importance analysis for the LSTM model with sliding window size of 20 frames. Thus, we utilized the gradient-based feature importance. The feature importance analysis reveals that drop width estimation is strongly influenced by the drop length, followed by the height of the drop’s center and velocity (Fig. 6a). The velocity of the drop is an important factor as it determines the kinetic energy of the drop which affects the deformation and spreading of the drop. The drop length and the height of the drop play a crucial role in determining the drop width. The lower importance of other variables may be attributed to their high correlation with these primary features (SI-Correlation matrix).

Our main goal is to ascertain the significance of features to implement the developed model on external data. Hence, we visualized the data abundance of our dataset (Fig. 6b**, Violin graphs**). In the data abundance diagram, each point corresponds to an existing frame in our dataset, associated with a particular value on the y-axis. For example, in the drop length diagram, the concentration of data was observed from 0.42 cm to 0.60 cm. However, there were also data available within the range of 0.35–0.75 cm as well. Considering the significant role of drop length in width estimation, we suggest using drops that are between 0.35 and 0.75 cm length range as the model input to ensure high precision. In the same line, the drop center height shall be from 0.07 to 0.115 cm and the velocity span from ~ 0 up to 35 cm/s. The feature importance and data abundance analysis indicates that the developed LSTM method estimates the drop width with a negligible error. However, this negligible error value can only be realized when the main features of new measurements fall within the trained data range specified above. The model trained is expected to keep its accuracy on new surfaces that have physical defects, as long as the range of side-view measurements falls within the range we have reported. Please note, the model's ability to accurately estimate drop width out of the given range has not been investigated.

## Conclusions

Including the temporal dependencies is critical for accurate estimations of drop width, when there is a defect on the surface that impedes drop motion. Our evaluation revealed that the LSTM model surpassed the competing models in terms of accuracy. With a result of 67.6 μm for the RMSE, the LSTM model outperformed the RF model, which achieved a best result of 109.0 μm. Taking into account the full range of drop width measurements in our dataset, which spans from a minimum of 1.6 mm to a maximum of 4.4 mm, the RMSE of 67 µm achieved by the LSTM model translates to an error percentage of 2.4%. The performance of the LSTM model eliminates the requirement for extra equipment while facilitating precise estimation along the entire sliding path. Thus the estimation of the drop width allows for a comprehensive analysis of drop behaviour.

Furthermore, the higher error observed in the RF model was attributed to the presence of relatively large defects on the surface. On smooth and homogeneous samples without defects, one can use a simple and interpretable regression algorithm like RF to determine drop width.

We've taken steps to enhance the model's versatility by exploring variations in drop viscosity and surface chemistry. As a result, the model's primary strength is its suitability for researchers working under similar conditions. We emphasize the importance of our sensitivity analysis section, which indicates boundaries for our model’s applicability in various research endeavours.

In essence, the proposed models offer a novel approach for estimating front-view drop width solely based on side-view measures. This approach simplifies sliding drop analysis significantly and enables researchers to measure drop width along the entire sliding path. The latter is experimentally unattainable with an optical setup. According to our findings, the RF model is suitable for flat samples, while the LSTM model is preferable for samples with defects. As we pioneer this research field, we employed the most known models to establish a foundational framework for future research.

As next steps, to improve precision, generality, and stability in future research, collecting more extensive datasets and using more sophisticated models like transformers could be beneficial. The superior modelling features of transformers might unlock a more profound insight into the time series' temporal patterns. Moreover, the direct analysis of drop videos through the use of Convolutional Neural Networks (CNNs) might be beneficial.

### Supplementary Information

Supplementary Information.

## Data Availability

The supporting materials and data generated and analysed during this study are included in this published article. The dataset: The “Dataset.xlsx” represents the dataset we compiled after processing and integrating the sliding drop videos. Training and validation process: The "Training and validation process.ipynb" file provides a detailed, step-by-step explanation of how we trained the LSTM model with a 20-slide window, which was determined to be the best model based on RMSE. LSTM learning process: The "LSTM learning process.xlsx" file includes a representation of the learning process for the LSTM model utilizing a 20-slide window. LSTM weights: The "LSTM weights.h5" file represents the fully trained 20-slide window LSTM model that can be employed by others for the purpose of estimating drop width in a same condition.
