# Supplementary material for: Estimating sliding drop width via side-view features using recurrent neural networks
Source: Sci Rep. 2024 May 27;14:12033. doi: 10.1038/s41598-024-62194-w (PMC11128450; doi:10.1038/s41598-024-62194-w)
Supplement: Supplementary file 1 — Supplementary Information. [file 41598_2024_62194_MOESM1_ESM.zip › Supplementary/Supplementary information.docx]

Estimating sliding drop width via side-view features using recurrent neural networks

Sajjad Shumaly^1^, Fahimeh Darvish^1^, Xiaomei Li^1^, Oleksandra Kukharenko^1^, Werner Steffen^1^, Yanhui Guo^2^, Hans-Jürgen Butt^1^, Rüdiger Berger^1^*

^1^Max Planck Institute for Polymer Research, Ackermannweg 10, D-55128, Mainz, Germany

^2^Department of Computer Science, University of Illinois Springfield, Springfield, IL, USA

* Corresponding Author. Email: berger@mpip-mainz.mpg.de

KEYWORDS: Sliding drop, Drop width estimation, Multivariate sequence analysis, Convolutional neural network (CNN), Long short-term memory (LSTM), Gated recurrent unit (GRU), bidirectional LSTM (BiLSTM), ConvLSTM

**RNNs’ architectures.**

RNNs maintain a hidden state that acts as a memory (h), allowing them to capture and remember information from previous elements in the sequence (**Figure 1a**). It takes input at each time step, updates the hidden state, and produces an output. The RNN cell's formula is as follows:

$h_{t}=tanh (W^{hx}x_{t}+W^{hh}h_{t-1}{+b}^{h})$ (1)

Where t denotes the time step, $x_{t}$denotes the current input,$W$ is the weight, $b$ is the bias, $h_{t-1}$ and $h_{t}$ denote the output of the last RNN cell, and current output, respectively (**Figure 1b**). The network's parameters, including weights and activation functions, are shared across all steps, enabling it to model sequential dependencies and relationships in the data. Learnable parameters, including weights and biases, determine how information is combined.

The LSTM cell's formula is as follows:

$f_{t}=\sigma(W^{f}\left[ h_{t-1},x_{t} \right]+b^{f})$ (2)

$i_{t}=\sigma(W^{i}\left[ h_{t-1},x_{t} \right]+b^{i})$ (3)

${c̃}_{t}=tanh(W^{c}\left[ h_{t-1},x_{t} \right]+b^{c})$ (4)

$c_{t}=f_{t} . c_{t-1}+ i_{t} .{c̃}_{t}$ (5)

$o_{t}=\sigma\left( W^{o}\left[ h_{t-1},x_{t} \right]+b^{o} \right)$ (6)

$h_{t}=o_{t}.tanh{(c}_{t})$ (7)

where $c_{t-1}$, and $c_{t}$ denote last cell state, and current cell state, ${c̃}_{t}$ denotes candidate cell state, $f_{t}$ denotes forget gate value, $i_{t}$ denotes update gate value, $o_{t}$ denotes output gate value, the operator ‘·’ denotes the pointwise multiplication of two vectors (**Figure 1c**).

The GRU cell's formula is as follows:

$z_{t}=\sigma(W^{z}\left[ h_{t-1},x_{t} \right]+b^{z})$ (8)

$r_{t}=\sigma(W^{r}\left[ h_{t-1},x_{t} \right]+b^{r})$ (9)

${h̃}_{t}=tanh(W^{h}\left[ {r_{t}. h}_{t-1},x_{t} \right]+b^{h})$ (10)

$h_{t}= z_{t} . {h̃}_{t}+\left( 1-z_{t} \right) . h_{t-1}$ (11)

Where $z_{t}$ denotes the update gate value, and $r_{t}$ denotes the reset gate value (**Figure 1d**).
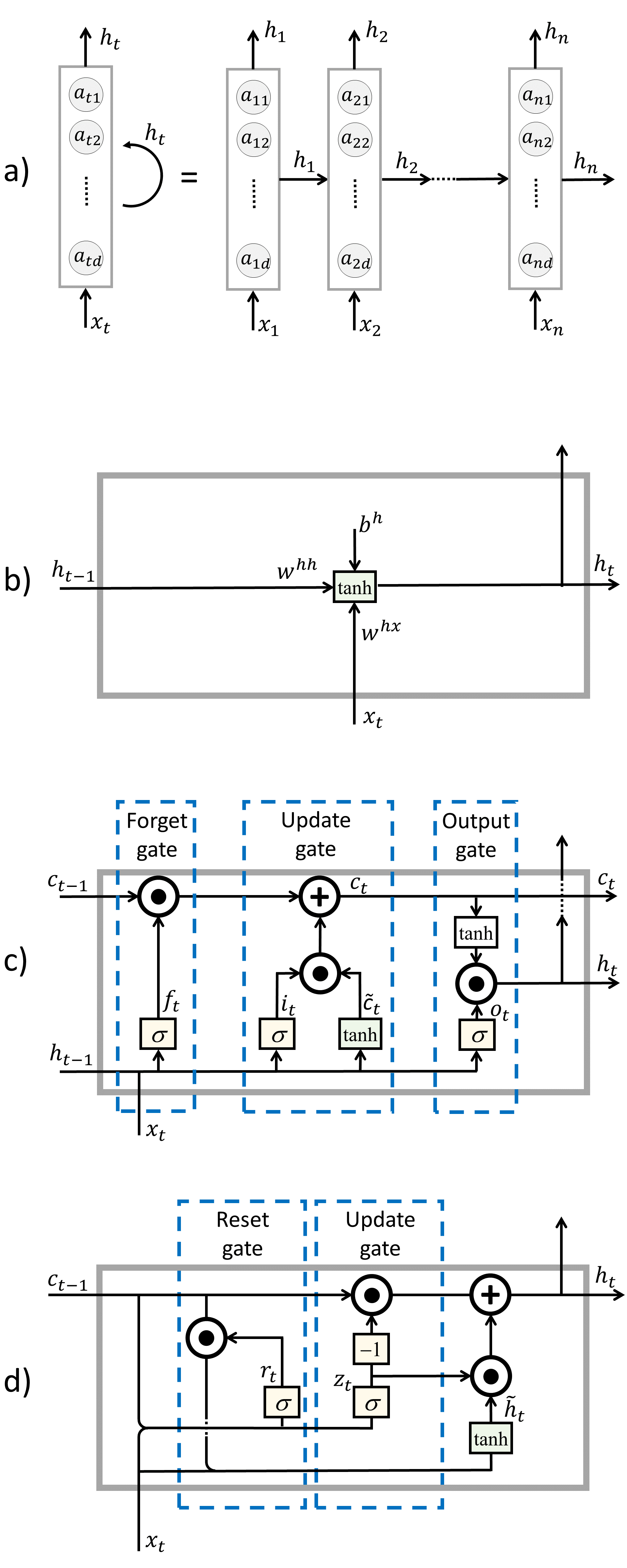


**Figure 1.** Visualizing the structure of RNN, LSTM, and GRU. a) Recurrent architecture representation. Left is shorthand notation and right is unfolded notation for RNNs. The d is the number of nodes that is a hyper-parameter. b) A vanilla RNN cell's architecture. The figure provides a closer examination of an individual cell within the RNN architecture. c) An LSTM cell's architecture. d) A GRU cell's architecture.

**Data distribution.** We've gathered a dataset that includes both samples with defects and defect-free samples (**Figure 2**). To enhance the model's generality, the defects have varying geometries in each type. The defects themselves were generated using SU8 on silicon samples, as explained in the manuscript. Finally, all samples containing defects have been coated with PFOTS. For the defect-free samples, we've used different surfaces and applied various coatings.

Samples with defects:

- Block defect-I geometry: thickness = 800 µm, width = 1000 µm, height = 106 µm.
- Block defect-II geometry: thickness = 800 µm, width = 2000 µm, height = 74 µm.
- Block defect-III geometry: thickness = 800 µm, width = 3000 µm, height = 174 µm.
- Block defect-III geometry: thickness = 800 µm, width = 3000 µm, height = 23.0 µm.

(The final validation sample)

- Cylindrical defect-I geometry: diameter = 800 µm, height = 31 µm.
- Cylindrical defect-II geometry: diameter = 800 µm, height = 47 µm.
- Step defect geometry: thickness = 800 µm, width = 2 cm, height = 30 µm.

Samples without defects:

- Thiols_Au: A perfluorodecanethiol monolayer on gold coated glass.
- PFOTS-Si: A 1H,1H,2H,2H-perﬂuoroctyltrichlorosilane coated silicon wafer sample.
- Glycerol on Thiols_Au: Varying glycerol concentrations ranging from 20% to 40% slid on Thiols_Au samples.
- Others: Twelve videos were recorded featuring various samples, including PFOTS coating on SiO2 and gold coating on glass.


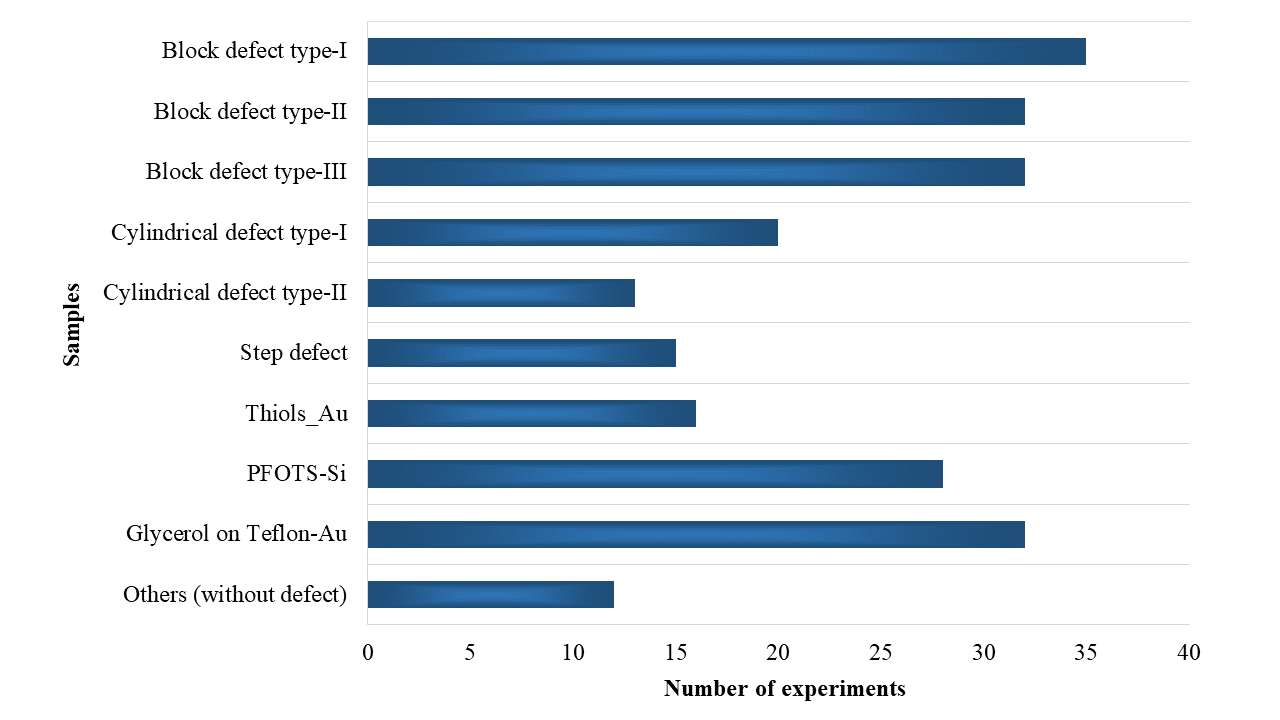


**Figure 2.** Data distribution.

**Correlation matrix.** The correlation matrix computed with Pearson correlation coefficients serves as a tool for quantifying and illustrating the linear relationships among the variables under investigation.

**Table 1.** The correlation matrix of variables.

|  | $\boldsymbol{\theta}_{\boldsymbol{a}}$ | $\boldsymbol{\theta}_{\boldsymbol{r}}$ | **Drop length** | **Drop center height** | **Velocity** | **Middle angle degree** |
| --- | --- | --- | --- | --- | --- | --- |
| $\boldsymbol{\theta}_{\boldsymbol{a}}$ | **1.00** | 0.19 | -0.44 | 0.48 | 0.12 | -0.06 |
| $\boldsymbol{\theta}_{\boldsymbol{r}}$ | 0.19 | **1.00** | -0.76 | 0.48 | 0.08 | 0.75 |
| **Drop length** | -0.44 | -0.76 | **1.00** | -0.68 | 0.09 | -0.67 |
| **Drop center height** | 0.48 | 0.48 | -0.68 | **1.00** | -0.14 | 0.48 |
| **Velocity** | 0.12 | 0.08 | 0.09 | -0.14 | **1.00** | -0.23 |
| **Middle angle degree** | -0.06 | 0.75 | -0.67 | 0.48 | -0.23 | **1.00** |

Data Availability

The supporting materials and data generated and analysed during this study are included in this published article.

- The dataset

The “Dataset.xlsx” represents the dataset we compiled after processing and integrating the sliding drop videos. In this dataset, the "Status" column indicates whether a video is associated with training, testing, or final validation measurements. Initially, we made random selections for these assignments but later maintained consistency across all algorithms to ensure a fair comparison. It's worth noting that the final validation records differ from the regular validation records. After dividing the dataset into testing and training subsets, we further split the training data into the typical training and validation sets for the training process. The final validation involves measurements conducted externally to the dataset, serving to assess the model's validity.

- Training and validation process

The "Training and validation process.ipynb" file provides a detailed, step-by-step explanation of how we trained the LSTM model with a 20-slide window, which was determined to be the best model based on RMSE. Using this file, reviewers can access the code, variables, and hyperparameters for examination. Furthermore, the document demonstrates how we utilized the trained model to incorporate the final validation metrics and estimate drop width. Ultimately, this file will be uploaded to GitHub and made freely accessible to everyone.

- LSTM learning process

The "LSTM learning process.xlsx" file includes a representation of the learning process for the LSTM model utilizing a 20-slide window. The learning process is based on its loss (MSE). Also MAE metric during the learning process is accessible.

- LSTM weights

The "LSTM weights.h5" file represents the fully trained 20-slide window LSTM model that can be employed by others for the purpose of estimating drop width in a same condition.
